# Long Non-Coding RNAs in Human Disease: An Overview of Biogenesis, Molecular Mechanism and Therapeutic Opportunities

**DOI:** 10.3390/cimb48040414

**Published:** 2026-04-17

**Authors:** Arvind Kumar Dubey, Anil Kumar, Zhadyrassyn Nurbekova, Navin Kumar

**Affiliations:** 1 Department of Agronomy and Horticulture, University of Nebraska-Lincoln, Lincoln, NE 68588, USA; arvindbiotech28@gmail.com; 2 Departamento de Biología Molecular de Plantas, Instituto de Biotecnología, Universidad Nacional Autónoma de México (UNAM), Cuernavaca 04510, Mexico; anilksp1986@gmail.com; 3 Department of Biotechnology and Microbiology, L.N. Gumilyov Eurasian National University, Astana 010000, Kazakhstan; zhadyrassyn.nurbekova@gmail.com; 4 Institute of Soil, Water, and Environmental Sciences, Agricultural Research Organization, Volcani Center, Rishon Lezion 7505101, Israel

**Keywords:** long non-coding RNAs, gene regulation, disease and mechanism, therapeutic targeting, diagnostic biomarkers

## Abstract

LncRNAs, defined as transcripts longer than 200 nucleotides with limited protein-coding potential, have emerged as important regulators of gene expression across multiple levels of cellular regulation. These molecules influence chromatin organization, transcriptional activity, and post-transcriptional processes through diverse interactions with DNA, RNA, and protein complexes. Although initially considered transcriptional byproducts, accumulating evidence now indicates that lncRNAs participate in a wide range of physiological processes and are implicated in numerous human diseases, including cancer, cardiovascular disorders, neurological diseases, and immune related conditions. However, the strength of mechanistic evidence varies substantially across the field, with robust functional validation currently limited to a relatively small number of well-characterized lncRNAs. In many cases, proposed regulatory roles remain supported primarily by expression correlations or limited perturbation studies, highlighting the need for careful evaluation of reproducibility, context dependence, and locus-specific effects. In addition, translating lncRNA discoveries into therapeutic strategies faces several practical challenges, including efficient tissue-specific delivery, subcellular localization constraints, isoform complexity, and potential off-target effects. This review provides an overview of current knowledge on lncRNA classification, biogenesis, and molecular mechanisms, evaluates their roles in human disease, and discusses emerging therapeutic approaches in the context of translational feasibility. By integrating mechanistic insights with current limitations and unresolved questions, we highlight priorities for future research aimed at harnessing lncRNAs for diagnostic and therapeutic applications in precision medicine.

## 1. Introduction

Long non-coding RNAs (lncRNAs) represent a substantial fraction of the eukaryotic transcriptome and comprise a highly diverse class of regulatory RNA molecules typically defined as transcripts longer than 200 nucleotides that lack protein-coding potential. Advances in transcriptome profiling have revealed that the majority of the human genome is pervasively transcribed, producing thousands of lncRNA transcripts with diverse regulatory functions [1,2,3].

LncRNAs are characterized by properties generally less robust than those of protein-coding mRNAs. Their transcripts are shorter, less stable, and expressed at levels approximately 10-fold lower, with expression patterns that are often tissue and cell type-specific. Single-cell analyses further reveal that lncRNA transcription is highly variable between individual cells, with burst frequencies and sizes significantly lower than those of mRNAs [4]. A key challenge in the field is distinguishing functional lncRNAs from transcriptional noise. Much of the human genome is transcribed, yet a considerable fraction of these transcripts are rapidly degraded by the RNA exosome and may represent unstable by-products of low-specificity RNA polymerase II initiation. This likely inflates estimates of the total number of functional lncRNA loci—a concern that grows as deeper RNA-sequencing of diverse tissues continues to expand the lncRNA catalog [5].

One approach to address this is the replication principle: lncRNAs consistently detected across multiple independent samples are considered higher-confidence candidates. Despite this, even low-confidence, rarely transcribed lncRNAs may reflect biologically meaningful events, such as transcription at sites of DNA damage repair or chromatin reorganization during the cell cycle, suggesting that transient transcription itself can serve functional roles [6,7].

Recent advances in high-throughput sequencing and functional genomics have revealed that lncRNAs are involved in the pathogenesis of a wide range of diseases, such as cancer, cardiovascular disorders, neurological conditions, and metabolic syndromes [8,9]. Dysregulation of lncRNA expression can disrupt cellular homeostasis by altering chromatin states, interfering with transcription factors, modulating mRNA stability, and acting as competitive endogenous RNAs (ceRNAs) that sequester microRNAs [10,11]. Mechanistically, lncRNAs participate in a broad spectrum of regulatory processes, including chromatin remodeling, transcriptional control, RNA processing, and post-transcriptional gene regulation. Many nuclear lncRNAs modulate chromatin states by recruiting epigenetic modifiers such as Polycomb repressive complexes or histone methyltransferases to specific genomic loci, thereby influencing transcriptional programs [2]. In the cytoplasm, lncRNAs can regulate mRNA stability, translation, and protein activity, and may act as competing ceRNAs that modulate microRNA (miRNA) availability [10]. This functional versatility enables lncRNAs to act as integrative regulatory hubs linking transcriptional, epigenetic, and post-transcriptional regulatory pathways.

Accumulating evidence indicates that dysregulation of lncRNA expression contributes to the pathogenesis of numerous human diseases. Aberrant expression of lncRNAs has been implicated in cancer progression, cardiovascular diseases, neurological disorders, and metabolic syndromes [1,8,9]. For example, lncRNAs such as Metastasis-Associated Lung Adenocarcinoma Transcript 1 (MALAT1), HOX Transcript Antisense RNA (HOTAIR), Nuclear paraspeckle assembly transcript 1 (NEAT1), and Antisense Noncoding RNA in the INK4 Locus (ANRIL) have been shown to influence tumor growth, metastasis, and cellular proliferation through epigenetic regulation and transcriptional control. Importantly, many lncRNAs exhibit strong tissue and cell-type-specific expression patterns, suggesting that they may serve as sensitive biomarkers for disease diagnosis and prognosis [12]. These properties have stimulated growing interest in exploring lncRNAs as potential therapeutic targets.

Despite the rapid expansion of lncRNA research, several fundamental challenges remain in understanding their biological functions and clinical relevance. Many lncRNAs display limited evolutionary conservation and exhibit complex isoform structures, complicating functional characterization across species. In addition, discrepancies between RNA interference-based knockdown experiments and CRISPR-mediated genomic perturbations have highlighted potential experimental artifacts and raised questions regarding whether observed phenotypes arise from the RNA transcript itself or from regulatory elements within the genomic locus [8]. Addressing these challenges is essential for establishing robust mechanistic frameworks that can support translational applications of lncRNA biology.

Recent technological advances, including high-throughput sequencing, single-cell transcriptomics, and improved RNA-targeting strategies, have substantially accelerated the discovery and functional characterization of lncRNAs. In parallel, progress in antisense oligonucleotide (ASO), RNA interference (RNAi), and CRISPR-based technologies has opened new opportunities to therapeutically modulate lncRNA expression and activity in vivo [7]. However, important obstacles remain, including efficient delivery, isoform specificity, and the identification of reliable biomarkers for monitoring therapeutic responses.

Although the number of annotated lncRNAs and disease associations has expanded rapidly, the strength of supporting evidence varies substantially across the field. Mechanistic understanding is robust for a relatively small number of lncRNAs, whereas many reported functions remain based primarily on expression correlations, knockdown phenotypes, or inferred molecular interactions. In addition, discrepancies between RNA interference-based studies, antisense approaches, and CRISPR-mediated perturbation have revealed important reproducibility and interpretation challenges. These issues highlight the need for a more critical framework that distinguishes well-supported mechanisms from preliminary or context-dependent observations.

**Figure 1:**
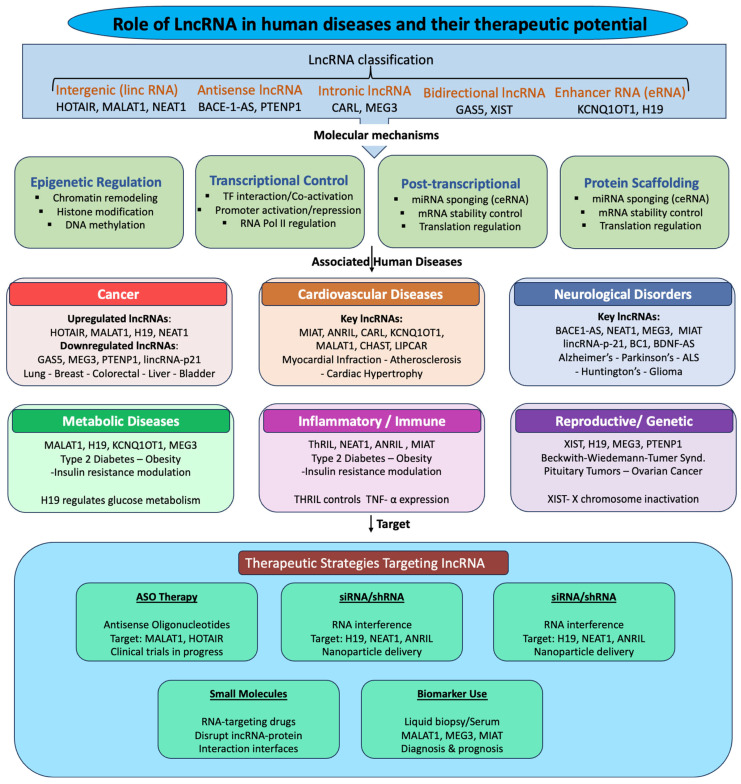
Schematic diagram of lncRNA origin, molecular mechanisms, disease associations, and therapeutic strategies.

In this review, we provide an overview of updated synthesis of the current understanding of lncRNA biology, focusing on their classification, molecular mechanisms of action, and emerging roles in human disease. Importantly, we critically evaluate the strength of existing evidence, highlight unresolved questions in the field, and discuss the practical challenges associated with targeting lncRNAs for therapeutic purposes. By integrating recent mechanistic and translational advances, this review aims to provide a conceptual framework for understanding how lncRNAs contribute to gene regulatory networks and how these molecules may be harnessed for future biomedical applications (Figure 1).

The strength of functional evidence supporting lncRNA-mediated regulatory mechanisms varies widely across studies. To provide a more rigorous framework for interpretation, lncRNA functions can be evaluated along a continuum of experimental validation, ranging from expression-based associations and computational predictions to in vitro perturbation studies, locus-resolved genetic analyses, and in vivo functional validation. While many lncRNAs have been implicated in disease based on differential expression or knockdown/overexpression experiments, such approaches may not distinguish between RNA-dependent effects and those arising from underlying genomic elements, transcriptional activity, or chromatin context. In contrast, locus-specific perturbation strategies, including CRISPR-mediated deletions or transcriptional interference, provide more definitive evidence of functional mechanisms. Throughout this review, we emphasize the importance of distinguishing between correlative and causally supported roles of lncRNAs.

## 2. Literature/Review Search Strategies

**Figure 2:**
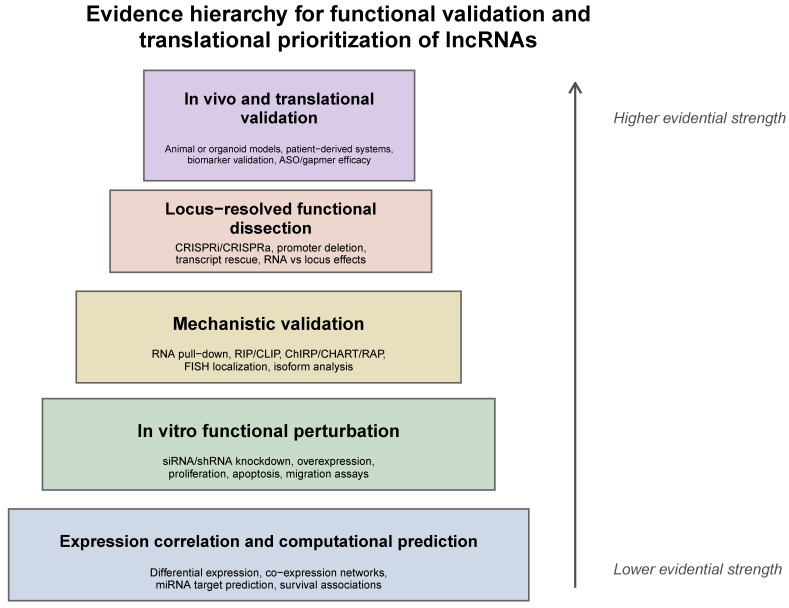
Evidence hierarchy for functional validation and translational prioritization of lncRNAs.

To ensure comprehensive and balanced coverage of the field, the literature for this review was retrieved from major scientific databases, including PubMed, Web of Science, and Scopus, using combinations of keywords such as *“long non-coding RNA,” “lncRNA mechanisms,” “lncRNA therapeutics,”* and *“lncRNA disease association.”* Additional references were identified through community-specific resources and by examining recent influential studies to capture emerging trends in lncRNA research. Studies were prioritized based on (i) strong mechanistic evidence supported by loss or gain-of-function experiments, (ii) clinical relevance demonstrated through patient datasets or clinical investigations, and (iii) recent advances in therapeutic targeting strategies. Representative lncRNAs, including HOTAIR, MALAT1, NEAT1, XIST, ANRIL, and PVT1, were selected as illustrative examples because they represent well-characterized functional classes and regulatory mechanisms. Emphasis was placed on citing scientifically rigorous and widely recognized studies to ensure an accurate and balanced synthesis of current knowledge (Figure 2).

## 3. Biogenesis and Classification of lncRNAs

### 3.1. Biogenesis of lncRNAs

**Figure 3:**
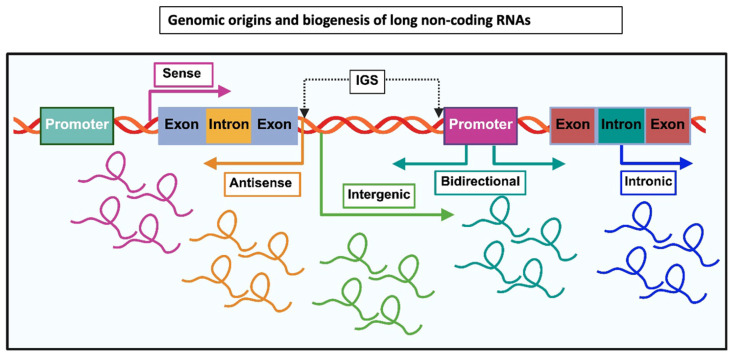
Schematic representation of genomic origins, biogenesis of LncRNAs and molecular mechanism of LncRNA. LncRNAs are broadly categorized into five classes based on their genomic origin: **sense lncRNAs** (overlapping coding exons/introns on the same strand), **antisense lncRNAs** (transcribed from the opposite strand of coding loci), **intergenic lncRNAs/lincRNAs** (arising from independent promoters in intergenic regions), **bidirectional lncRNAs** (transcribed divergently from shared or adjacent promoters near coding genes), and **intronic lncRNAs** (originating entirely within intronic sequences). All classes are transcribed by RNA Polymerase II and undergo 5′ capping, splicing, and 3′ polyadenylation. Arrows indicate transcriptional direction; blue and orange bars represent protein-coding and lncRNA exons, respectively. Created in BioRender. Kumar, N. (2026) https://BioRender.com/xv4692x.

The biogenesis of lncRNAs shares several features with the transcription and processing of protein-coding genes, yet also displays distinct regulatory characteristics. Most lncRNAs are transcribed by RNA polymerase II and undergo canonical RNA processing events, including 5′ capping, splicing, and 3′ polyadenylation [7,13]. However, lncRNA transcription is often less efficient than that of protein-coding genes, resulting in lower expression levels and greater cell-type specificity. LncRNA genes frequently originate from diverse genomic elements such as promoters, enhancers, bidirectional promoters, and pseudogene loci. In some cases, transcription of lncRNAs occurs from enhancer regions, generating enhancer RNAs that participate in transcriptional activation [14]. These diverse origins contribute to the complex regulatory architecture of lncRNA loci. Alternative splicing is highly prevalent among lncRNAs and frequently generates multiple transcript isoforms from a single genomic locus. In addition to canonical splicing, lncRNAs may also undergo post-transcriptional modifications such as adenosine-to-inosine (A-to-I) RNA editing and N6-methyladenosine (m6A) methylation, which influence RNA stability, localization, and function [15]. Following transcription, lncRNAs exhibit distinct subcellular localization patterns that are closely linked to their biological functions. Many lncRNAs remain in the nucleus, where they associate with chromatin or nuclear bodies such as paraspeckles (e.g., NEAT1). Others are exported to the cytoplasm through RNA export pathways involving transport factors such as NXF1-NXT1, where they participate in post-transcriptional regulation of gene expression [16]. Together, the complex biogenesis, diverse genomic origins, and multifaceted regulatory mechanisms of lncRNAs highlight their central role in gene regulatory networks and underscore the need for integrative approaches to fully understand their biological functions (Figure 3).

### 3.2. Classification of lncRNAs

LncRNAs represent a highly heterogeneous class of transcripts that differ widely in genomic origin, molecular mechanisms, and functional roles. Because lncRNAs lack conserved open reading frames and often exhibit low sequence conservation, classification based solely on sequence features is challenging. Consequently, several complementary classification frameworks have been proposed based on genomic context, molecular mechanism, and subcellular localization [6,17]. Among these, classification according to genomic location relative to protein-coding genes remains the most widely used because it provides insight into transcriptional regulation and potential regulatory relationships with neighboring genes [18].

Most lncRNAs are transcribed by RNA polymerase II and undergo processing events similar to messenger RNAs, including 5′ capping, splicing, and polyadenylation [12]. However, compared with protein-coding transcripts, lncRNAs generally exhibit lower expression levels, greater tissue specificity, and more dynamic transcriptional regulation. LncRNA loci frequently originate from promoters, enhancers, introns, or intergenic regions, reflecting their diverse regulatory origins and functions. Importantly, emerging studies indicate that genomic context not only determines transcriptional regulation but may also influence the regulatory mechanisms through which lncRNAs act in cis or trans (Figure 3).

#### 3.2.1. Based on Genomic Location Relative to Protein-Coding Genes

LncRNAs are most widely categorized based on their genomic location relative to protein-coding genes: sense, antisense, bidirectional, intronic, and intergenic (Figure 3) [18]. Sense lncRNAs: Transcribed from the same strand as a protein-coding gene; can overlap exons or introns. Antisense lncRNAs: Transcribed from the opposite strand of a protein-coding gene. Intronic lncRNAs: Derived entirely from introns of protein-coding genes. Long-Intergenic lncRNAs (lincRNAs): Located between protein-coding genes (in intergenic regions). Bidirectional lncRNAs: Transcribed in the opposite direction to a nearby protein-coding gene, often sharing a promoter (<1 kb apart). Enhancer-associated lncRNAs (eRNAs): Transcribed from enhancer regions [13,17].

##### Sense lncRNAs

Sense lncRNAs are transcribed from the same genomic strand as protein-coding genes and may share exonic or intronic regions with their corresponding transcripts [19]. Because of this positional relationship, sense lncRNAs can influence gene expression through transcriptional interference, modulation of RNA splicing, or direct RNA–RNA interactions with overlapping Dysregulation of sense lncRNAs has been linked to a variety of human diseases, most notably cancer, where it contributes to abnormal gene expression and progression.

##### Antisense lncRNAs

Antisense lncRNAs are transcribed from the opposite strand of protein-coding genes and often overlap exonic or intronic regions, resulting in natural antisense transcripts. These transcripts, often referred to as natural antisense transcripts, can regulate gene expression through multiple mechanisms, including chromatin remodeling, transcriptional interference, and RNA–RNA duplex formation that influences mRNA stability, splicing, or translation [20,21].

Because of their overlapping genomic arrangement, antisense lncRNAs frequently act in cis to regulate the expression of their corresponding sense genes. However, several antisense lncRNAs have also been shown to exert trans-regulatory effects on distant genes. Increasing evidence links antisense lncRNAs to diverse pathological conditions, including cancer, neurological disorders, and cardiovascular diseases.

##### Intronic lncRNAs

Intronic lncRNAs are entirely transcribed from protein-coding genes’ intronic regions, which do not overlap with exons. These transcripts may arise either from independent transcriptional units within introns or from processing of intronic RNA sequences. They can regulate gene expression in both cis and trans by being independently transcribed or processed from intronic sequences. Intronic lncRNAs are involved in a variety of regulatory processes, including transcriptional control, alternative splicing, chromatin modification, and other cellular responses [22]. Some intronic lncRNAs influence the expression of their host genes, while others affect distant targets via RNA-protein interactions. Dysregulation of intronic lncRNAs has been linked to cancer, metabolic disorders, and neurodegenerative diseases, highlighting their functional significance in human pathology.

Some intronic lncRNAs regulate the expression of their host genes, whereas others act on distant targets by interacting with regulatory proteins or RNA molecules. Emerging studies suggest that intronic lncRNAs participate in diverse biological processes and that their dysregulation contributes to diseases such as cancer and metabolic disorders.

##### Intergenic lncRNAs

LincRNAs are a type of lncRNA that is transcribed from genomic regions located between protein-coding genes and therefore do not overlap with coding sequences. Unlike intronic or antisense lncRNAs, lincRNAs do not overlap with coding sequences and thus function independently [12].

Functionally, lincRNAs have been shown to regulate gene expression through diverse mechanisms, including recruitment of chromatin-modifying complexes, modulation of transcription factor activity, and regulation of RNA stability. Several well-characterized lincRNAs act as molecular scaffolds that facilitate the assembly of regulatory protein complexes, thereby coordinating epigenetic and transcriptional control. Due to their strong tissue-specific expression patterns, many lincRNAs are currently being investigated as potential biomarkers and therapeutic targets.

##### Bidirectional lncRNAs

Bidirectional lncRNAs are transcribed from the promoter regions located close to protein-coding genes but in the opposite direction, typically within a distance of less than 1 kilobase, using the same promoter. This arrangement enables coordinated regulation of both the lncRNA and its neighbouring gene, which frequently affects transcriptional activity, chromatin remodelling, and RNA stability. Bidirectional lncRNAs can function as enhancers, decoys, or scaffolds, influencing gene expression in either cis or trans. They are involved in a variety of biological processes, including cell differentiation, proliferation, and stress responses. Dysregulation of bidirectional lncRNAs has been linked to diseases like cancer, neurodegeneration, and cardiovascular disorders, emphasizing their regulatory and therapeutic importance [23].

#### 3.2.2. Enhancer-Associated Long Non-Coding RNAs

Enhancer-associated lncRNAs, commonly referred to as eRNAs, are transcribed from active enhancer regions. These transcripts are typically short-lived, often non-polyadenylated, and frequently transcribed bidirectionally from enhancer loci marked by epigenetic signatures such as H3K27ac and H3K4me1 [14,24]. eRNAs are increasingly recognized as functional regulators of gene expression, facilitating enhancer–promoter looping and recruitment of transcriptional co-activators such as Mediator and cohesin. Through these mechanisms, eRNAs contribute to the activation of transcriptional programs associated with development, immune responses, and disease processes.

#### 3.2.3. Based on Molecular Mechanism of Action

lncRNAs can be classified by their mechanism, such as how they regulate gene expression. Decoy: Bind and titrate away proteins (e.g., transcription factors) or miRNAs. As an example, GAS5 lncRNA binds glucocorticoid receptor to repress gene expression. Guide: Recruit proteins (chromatin modifiers, transcription factors) to target loci. As an example, HOTAIR recruits PRC2 to silence the HOXD locus. Scaffold: Serves as a platform to assemble ribonucleoprotein complexes. As an example, TERC (telomerase RNA) forms a scaffold for telomerase assembly. Signal: Act as molecular signals integrating stimuli (developmental or environmental). As an example, lncRNA X-inactive specific transcript (XIST) mediates X-chromosome inactivation. Enhancer-like: Facilitates chromatin looping and enhancer-promoter interactions [9].

Besides these, lncRNAs can also be classified according to their subcellular localization, i.e., nuclear [XIST, NEAT1], cytoplasmic (NORAD, TINCR), or both [6]. lncRNAs can also be grouped based on functional role in diseases like: Oncogenic lncRNAs (Onco-lncRNAs): e.g., HOTAIR, MALAT1; Tumor suppressor lncRNAs: e.g., MEG3 (Maternally Expressed Gene 3); Disease-specific lncRNAs: linked to cardiovascular, neurodegenerative, or metabolic disorders [9]. Although this mechanistic framework is useful conceptually, it is important to note that many lncRNAs exhibit multiple mechanisms simultaneously, highlighting the complexity of lncRNA mediated regulatory networks.

## 4. Molecular Mechanisms of lncRNA Function

### 4.1. Transcriptional Regulation

**Figure 4:**
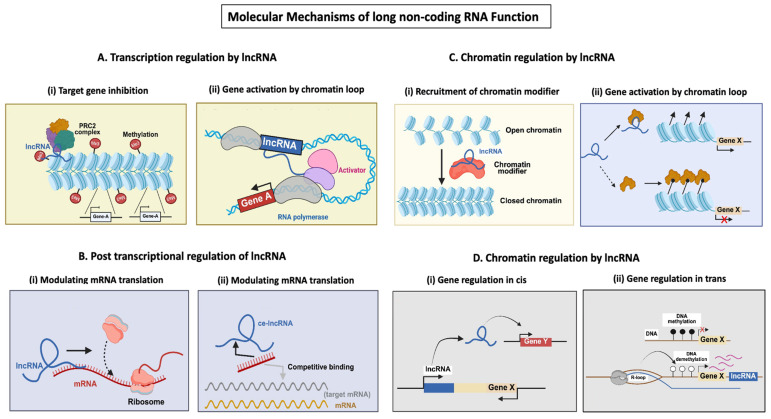
Molecular mechanism of LncRNA; LncRNAs modulate gene expression through four principal mechanisms: (**A**) Transcriptional regulation of LncRNA: (i) Target gene inhibition, recruitment of repressive chromatin complexes (e.g., PRC2) to target loci, inducing histone methylation (H3K27me3) and transcriptional silencing; (ii) Gene activation by chromatin loop, facilitation of promoter–enhancer looping to recruit RNA Polymerase II and drive transcriptional activation. (**B**) Post-transcriptional regulation of LncRNA: (i) Modulating mRNA translation, interaction with mRNAs or ribosomal components to modulate translational efficiency; (ii) Modulating mRNA translation, competitive sequestration of microRNAs (miRNA sponging/ceRNA activity), relieving miRNA-mediated repression of target mRNAs. (**C**) Chromatin regulation by LncRNAs, LncRNAs modulate chromatin architecture and gene expression through three principal mechanisms: (i) Acting as molecular guides or scaffolds to recruit chromatin-modifying complexes (e.g., histone methyltransferases, deacetylases) to specific genomic loci, inducing chromatin compaction or relaxation and consequent transcriptional repression or activation; (ii) Functioning as molecular decoys to sequester chromatin modifiers away from target genes, indirectly modulating their transcriptional status. (**D**) Chromatin regulation by LncRNAs: (i) and (ii) Directly associating with chromatin in *cis* to regulate neighboring genes or in *trans* to act at distant loci, involving RNA–DNA hybrid (R-loop) formation, modulation of DNA methylation, and alteration of local chromatin states. Together, these mechanisms underscore the versatile roles of lncRNAs in epigenetic and transcriptional gene regulation. Created in BioRender. Kumar, N. (2026) https://BioRender.com/xv4692x.

LncRNAs can act in cis or trans to modulate transcription (Figure 4A). HOTAIR, transcribed from the HOXC locus, represses transcription in the HOXD locus by recruiting PRC2 and LSD1 complexes [25]. Many lncRNAs are regulated by RNA polymerase II machinery, with transcription factors binding to their promoters and enhancers. Cell-type-specific transcription factors orchestrate their expression, e.g., p53 directly binds and activates *lincRNA-p21* in response to DNA damage [26]. Some lncRNAs are transcribed from enhancers, known as eRNAs. Super-enhancers drive robust lncRNA expression in certain contexts, such as development and cancer. The lncRNA *MALAT1* is regulated by super-enhancers in cancer cells [27]. Multiple promoters can produce isoforms with distinct functions or stabilities. Alternative splicing contributes to transcript diversity and regulation, e.g., *NEAT1* has two isoforms controlled by alternative 3′ processing [28]. Some lncRNAs are involved in the feedback regulation of their own transcription. Crosstalk with miRNAs can affect promoter usage and transcriptional repression. LncRNA loci are often associated with chromatin loops, bringing promoters and enhancers into proximity. Nuclear domains (e.g., paraspeckles) can sequester transcription factors, indirectly affecting lncRNA transcription, e.g., *NEAT1* is essential for paraspeckle formation, which in turn affects gene expression [29]. External stimuli (e.g., stress, hormones) activate signaling pathways (e.g., NF-κB, STAT3) that directly control lncRNA transcription. *Lethe* is an lncRNA induced by NF-κB signaling and negatively regulates NF-κB itself [30].

### 4.2. Post-Transcriptional Regulation

Post-transcriptional regulation determines lncRNA abundance, stability, localization, and function after they are transcribed (Figure 4B). LncRNAs can bind mRNA or microRNAs to affect splicing, stability, translation, or degradation. For example, MALAT1 regulates alternative splicing by modulating splicing factor activity [31]. LncRNAs often undergo alternative splicing, producing isoforms with different functions or stabilities, e.g., MALAT1 generates a mature lncRNA and a tRNA-like small RNA via RNase P cleavage. LncRNA’s stability is influenced by non-canonical polyadenylation sites or lack of polyA tails [8,32]. N6-methyladenosine (m6A) methylation regulates lncRNA degradation, nuclear export, and interactions, e.g., METTL3/14 methyltransferases catalyze m6A modification, and Readers like YTHDC1 bind methylated lncRNAs to modulate stability. Pseudouridylation, m5C, and A-to-I editing also shape lncRNA fate [33]. LncRNAs often accumulate in the nucleus or are exported to the cytoplasm via ALYREF–NXF1 pathways and CRM1 export receptor. Retention motifs (e.g., triple helix structures) anchor lncRNAs in nuclear compartments [34]. RNA-binding proteins (RBPs) such as HuR, AUF1, and IGF2BP stabilize or destabilize lncRNAs. Exosome and XRN2 mediate degradation of unstable transcripts [35]. LncRNAs are packaged into exosomes and microvesicles, influencing intercellular communication, and sorting involves RBPs (hnRNPA2B1, YBX1) and lipid raft-associated pathways. Although considered “non-coding,” some lncRNAs are translated into small functional peptides. This process depends on ribosome association and start codon selection [36].

### 4.3. Decoys and Scaffolds Functions

LncRNAs, as molecular decoys, bind and sequester specific proteins (e.g., transcription factors) or miRNAs away from their normal targets [Figure 4B(i,ii)]. This prevents these regulators from interacting with DNA, mRNA, or protein partners. LncRNAs act as miRNA Sponges and Protein Decoys. miRNA Sponges: lncRNAs contain miRNA response elements that competitively bind miRNAs, reducing repression of miRNA target mRNAs. Protein Decoys: lncRNAs directly bind proteins such as transcription factors or chromatin modifiers, sequestering them in the nucleus or cytoplasm. Growth arrest-specific transcript 5 (GAS5) binds the glucocorticoid receptor DNA-binding domain, preventing receptor association with DNA and repressing target genes. Phosphatase and Tensin Homolog Pseudogene 1 (PTENP1): acts as a decoy for miR-19b, protecting PTEN mRNA from degradation. NORAD: sequesters PUMILIO proteins, preventing destabilization of mRNAs important for genomic stability [37,38,39]. Some lncRNAs act as decoys for transcription factors or miRNAs. Taurine upregulated gene 1 (TUG1), for example, sponges miR-9 to modulate gene expression in glioma [40].

lncRNAs can act as molecular scaffolds, bringing together multiple proteins into ribonucleoprotein complexes that coordinate gene expression regulation. lncRNAs have modular domains enabling simultaneous binding to different effector proteins [41]. Scaffold lncRNAs can assemble chromatin remodelling complexes, histone modification enzymes, or transcriptional regulators at specific loci. HOTAIR binds PRC2 at its 5′ domain and LSD1/CoREST/REST complex at its 3′ domain, linking histone methylation and demethylation machinery to silence the HOXD locus. ANRIL assembles PRC1 and PRC2 to repress the INK4b-ARF-INK4a locus. NEAT1 scaffolds paraspeckle proteins (NONO, SFPQ) to form nuclear paraspeckles involved in gene regulation [41].

### 4.4. ceRNA/miRNA Sponging lncRNAs

LncRNAs can regulate gene expression at the post-transcriptional level by functioning as competing ceRNAs, a mechanism in which RNA molecules communicate through shared miRNA response elements (MREs) [Figure B(ii)]. In this framework, lncRNAs act as molecular “sponges” that sequester miRNAs, thereby reducing their availability to bind target mRNAs and relieving miRNA-mediated repression [42,43]. This interaction establishes a complex regulatory network involving lncRNAs, circular RNAs, pseudogenes, and mRNAs, collectively modulating gene expression dynamics in a context-dependent manner. The efficiency of miRNA sequestration depends on several factors, including relative transcript abundance, subcellular localization, and binding affinity, highlighting the quantitative and highly dynamic nature of ceRNA crosstalk [44].

This regulatory mechanism has been extensively implicated in disease pathogenesis, particularly in cancer, where dysregulated lncRNA–miRNA–mRNA networks contribute to tumor initiation, progression, and therapy resistance. For instance, the lncRNA HOTAIR functions as a sponge for miR-331-3p and miR-124, leading to the upregulation of oncogenic targets such as HER2 and EZH2 in gastric cancer [45,46]. Similarly, MALAT1 sequesters members of the miR-200 family, thereby promoting epithelial–mesenchymal transition (EMT) and metastasis across multiple cancer types [47]. Other examples include SNHG6, which sponges miR-6509-5p to enhance HIF1A expression in hepatocellular carcinoma, and NEAT1, which modulates glioma progression by targeting miR-449b-5p and regulating C-MET expression [48,49].

In contrast, several lncRNAs exert tumor-suppressive effects through miRNA sponging mechanisms. For example, MEG3 sequesters miR-21 and miR-141 in hepatocellular carcinoma and ovarian cancer, restoring the expression of tumor suppressors such as PTEN and PDCD4 and thereby inhibiting proliferation and inducing apoptosis [48]. Similarly, GAS5 acts as a sponge for miR-21 in triple-negative breast cancer, enhancing chemosensitivity by relieving repression of key tumor suppressor pathways [50]. The pseudogene-derived lncRNA PTENP1 also functions as a ceRNA by binding miR-19b, miR-20a, and miR-26a, thereby stabilizing PTEN expression and suppressing tumorigenesis [51]. Together, these findings underscore the dual role of lncRNA-mediated miRNA sponging in regulating oncogenic and tumor-suppressive pathways.

Beyond cancer, ceRNA-mediated regulation by lncRNAs also plays significant roles in non-malignant diseases. In cardiovascular disorders, the lncRNA CHRF acts as a sponge for miR-489, influencing myosin heavy chain expression and contributing to cardiac hypertrophy [52]. In neurodegenerative diseases, lncRNAs such as BDNF-AS regulate neuronal survival by modulating miRNA networks that control brain-derived neurotrophic factor (BDNF) expression. Additionally, lncRNAs including NEAT1 and TUG1 have been implicated in inflammatory and metabolic diseases through miRNA-dependent regulation of cytokine signaling and lipid homeostasis [53]. These observations highlight the broad relevance of ceRNA networks across diverse physiological and pathological contexts.

Despite extensive evidence supporting the ceRNA hypothesis, several conceptual and experimental challenges remain. Effective miRNA sponging requires sufficiently high expression levels of lncRNAs relative to their target miRNAs, a condition that may not be universally met in vivo. Furthermore, many reported ceRNA interactions are based on correlative expression analyses rather than direct functional validation. Distinguishing true ceRNA activity from indirect regulatory effects therefore requires rigorous quantitative and mechanistic approaches. Additionally, it remains unclear in many cases whether observed phenotypes are mediated by the RNA transcript itself, the act of transcription, or regulatory elements within the genomic locus.

Overall, lncRNA-mediated miRNA sponging represents a critical layer of post-transcriptional gene regulation with significant implications for human disease. Continued integration of high-throughput sequencing, computational modeling, and precise functional assays will be essential to fully elucidate ceRNA networks and to determine their translational potential as therapeutic targets.

To improve clarity, representative lncRNAs discussed in this review are annotated according to the strength of supporting evidence and whether their functional effects are RNA-dependent or locus-dependent.

### 4.5. Chromatin Remodelling and Epigenetic Regulation

The expression of lncRNAs is tightly controlled by epigenetic mechanisms, including chromatin remodelling, histone modifications, and DNA methylation (Figure 4C,D). Histone modifications and DNA methylation are interconnected epigenetic mechanisms that regulate gene expression by altering chromatin structure and accessibility, histone modifications act through covalent changes to histone tails, while DNA methylation typically involves the addition of a methyl group to cytosine residues at CpG dinucleotides, with both processes collectively orchestrating stable and heritable transcriptional programs without altering the underlying DNA sequence. Conversely, many lncRNAs can also recruit chromatin-modifying complexes to specific loci to regulate gene expression in cis or trans. LncRNAs can guide chromatin-modifying complexes to specific genomic loci, modulating gene expression. For instance, XIST recruits the polycomb repressive complex 2 (PRC2) to silence one of the X chromosomes in females [7,13]. The SWI/SNF (BAF), ISWI, CHD, and INO80 families remodel chromatin structure and thereby control lncRNA transcription [8].

DNA methylation at CpG islands in promoters suppresses lncRNA transcription. Hypomethylation can lead to aberrant activation of oncogenic lncRNAs. As an example, the *MEG3* lncRNA promoter is hypermethylated in many cancers, silencing its tumor-suppressive functions. DNA methyltransferases (DNMT1, DNMT3A/B) deposit methyl marks, while TET enzymes mediate demethylation [2,38].

Histone marks profoundly impact lncRNA loci transcription as Activating (H3K27ac: activate enhancers and promoters) or Repressive marks, e.g., *HOTAIR* interacts with PRC2 to deposit H3K27me3 across the HOXD locus, suppressing transcription, and XIST, essential for X-chromosome inactivation, recruits PRC2 to spread H3K27me3 over the inactive X chromosome [54,55]. Thus, chromatin remodelling regulates lncRNA expression, while lncRNAs in turn orchestrate chromatin states.

## 5. Role of lncRNAs in Human Diseases

LncRNAs have emerged as key regulatory molecules that influence gene expression through diverse and context-dependent mechanisms, thereby contributing to the onset and progression of a wide range of diseases [56]. Unlike protein-coding genes, lncRNAs exert their functions at multiple regulatory levels, including chromatin remodeling, transcriptional control, and post-transcriptional regulation, often acting as molecular scaffolds, guides, decoys, or competing endogenous RNAs. These mechanistic actions enable lncRNAs to modulate critical cellular processes such as proliferation, apoptosis, differentiation, and stress responses, which are frequently dysregulated in pathological conditions. Importantly, the functional impact of lncRNAs is highly cell-type and environment-specific, complicating their interpretation but also underscoring their potential as precise biomarkers and therapeutic targets. In the following sections, we discuss the roles of lncRNAs across major disease categories, highlighting how their underlying molecular mechanisms contribute to disease pathogenesis.

### 5.1. Cancer

LncRNAs contribute to cancer initiation and progression through multiple interconnected molecular mechanisms, including chromatin remodeling, transcriptional regulation, and post-transcriptional control. Many oncogenic lncRNAs promote tumorigenesis by recruiting epigenetic modifiers such as Polycomb repressive complexes to silence tumor suppressor genes, while others modulate transcription factor activity or alter enhancer–promoter interactions. At the post-transcriptional level, lncRNAs frequently function as ceRNAs, sequestering miRNAs and thereby stabilizing oncogenic mRNAs or repressing tumor suppressor pathways. Additionally, lncRNAs can influence key signaling pathways, including PI3K/AKT, Wnt/β-catenin, and TGF-β, thereby regulating proliferation, EMT, metastasis, and therapy resistance [57]. These mechanistic roles collectively underpin the widespread involvement of lncRNAs in diverse cancer types. It regulate gene expression at epigenetic, transcriptional, and post-transcriptional levels, playing crucial roles in cancer initiation, progression, metastasis, and therapy resistance (Table 1). LncRNAs such as HOTAIR, MALAT1, and PVT1 are overexpressed in various tumors and promote proliferation, metastasis, and therapy resistance [58]. As an epigenetic Regulator, lncRNAs recruit chromatin-modifying complexes (e.g., PRC2) to silence tumor suppressor genes, e.g., HOTAIR promotes metastasis in breast cancer by guiding PRC2. As a transcriptional Regulator, lncRNAs act as enhancers or repressors, e.g., LINC01133 inhibits EMT in colorectal cancer [49,59]. During Post-Transcriptional Regulation, lncRNAs function as competing ceRNAs, sponging miRNAs that stabilize mRNAs or modulate splicing, e.g., MALAT1 influences alternative splicing and metastasis. As a Signal Transduction Modulator, lncRNAs interact with kinases and signaling molecules, e.g., LINC00958 promotes hepatocellular carcinoma via the miR-3619-5p/HDAC1 axis [60].

PVT1 plays a significant role in different disease types, such as ovarian cancer, gastric cancer, lymphoma, etc. PVT1 leads tumor progression in ovarian cancer through several modifications, including EZH2-mediated silencing of miR-214, activation of the TGF-β pathway via the miR-148a-3p/AGO1 axis, and CTGF-mediated epithelial-to-mesenchymal transition—while also functioning as a YAP1-dependent stress-responsive lncRNA that promotes metastasis and resistance to platinum-based chemotherapy [61,62,63]. PVT1 is significantly overexpressed in gastric cancer tissues compared to adjacent normal mucosa, and its elevated expression independently predicts worse overall survival, correlating with deeper tumor invasion and advanced TNM stage via epigenetic silencing of the tumor suppressors p15 and p16 through EZH2 recruitment. Beyond its prognostic role, PVT1 is a central mediator of chemoresistance: it is upregulated in both cisplatin- and paclitaxel-resistant gastric cancer cell lines, and its silencing restores drug sensitivity by decreasing MDR1, MRP1, k-Ras, and c-Myc expression while increasing pro-apoptotic Bax levels and inducing G2/M cell cycle arrest [64,65]. PVT1, first identified at a translocation breakpoint in murine plasmacytomas and subsequently linked to immunoglobulin translocations in Burkitt lymphoma, is aberrantly overexpressed in diffuse large B-cell lymphoma (DLBCL), the most common and aggressive subtype of non-Hodgkin lymphoma, where high expression predicts poor prognosis [66].

**Table 1:** Some notable lncRNAs and their associated cancers.

LncRNAs	Cancer Type	Functions	References
HOTAIR	Breast, colorectal, lung	Epigenetic silencing, metastasis	[67,68,69]
MALAT1	Lung, liver, prostate	Splicing regulation, proliferation	[70,71,72]
PVT1	Ovarian, gastric, lymphoma	Oncogene activation, drug resistance	[65,73,74]
GAS5	Breast, prostate, leukemia	Tumor suppression, apoptosis induction	[75,76,77]
NEAT1	Pancreatic, glioma, leukemia	Inflammation, cell survival	[78,79,80]

These findings collectively position PVT1 as a compelling prognostic biomarker and a multi-pathway therapeutic target in ovarian cancer, with RNAi, antisense oligonucleotides, and CRISPR/Cas9-based silencing strategies currently under investigation.

Some notable lncRNAs and their associated cancers are given in Table 1.

### 5.2. Cardiovascular Diseases

In cardiovascular diseases, lncRNAs regulate cellular homeostasis through mechanisms that include epigenetic modulation, transcriptional control, and post-transcriptional regulation in vascular and cardiac cell types. lncRNAs can influence chromatin architecture by recruiting histone-modifying complexes, thereby controlling gene expression programs associated with vascular remodeling and cardiac hypertrophy [81]. At the transcriptional level, they interact with transcription factors and signaling pathways to regulate endothelial function and smooth muscle cell proliferation. Post-transcriptionally, lncRNAs modulate mRNA stability and translation or act as miRNA sponges, thereby influencing key pathways involved in inflammation, fibrosis, and contractile function. Through these mechanisms, lncRNAs play central roles in the development of atherosclerosis, myocardial infarction, and cardiac hypertrophy. Further, lncRNAs such as ANRIL and Myocardial Infarction Associated Transcript (MIAT) regulate vascular smooth muscle cell proliferation and myocardial infarction outcomes, implicating them in atherosclerosis and cardiac hypertrophy [81,82]. ANRIL (CDKN2B-AS1): regulates cell proliferation and inflammation in vascular smooth muscle cells. H19: promotes vascular smooth muscle cell proliferation and plaque instability. Mhrt: protects the heart against pathological hypertrophy by antagonizing Brg1-mediated chromatin remodelling. Chast: Promotes cardiac hypertrophy. MIAT: involved in ischemic injury and remodelling. LncRNA-CAIF: inhibits autophagy-mediated cardiomyocyte death by targeting p53. KCNQ1OT1: Regulates ion channels implicated in conduction disorders. GAS5 and MALAT1: Modulate endothelial dysfunction and vascular tone [81,82,83,84]. Thus, LncRNAs may be an emerging powerful regulator and biomarker in cardiovascular diseases. Their cell-type specificity and mechanistic diversity make them promising targets for innovative therapies. However, translating these findings into clinical applications requires: Better delivery systems for lncRNA modulation; Validation of safety and efficacy in large-scale studies; Improved understanding of their interactions with microRNAs and proteins.

### 5.3. Neurological Disorders

LncRNAs play critical roles in the nervous system by regulating gene expression through epigenetic, transcriptional, and post-transcriptional mechanisms that are essential for neuronal development, synaptic plasticity, and cellular homeostasis. Many lncRNAs influence chromatin states and transcriptional programs governing neuronal differentiation and function, while others regulate RNA processing, stability, and translation in response to neuronal activity [7]. In addition, lncRNAs frequently act as competing endogenous RNAs, modulating microRNA availability and thereby affecting pathways involved in neuroinflammation, protein aggregation, and oxidative stress. Dysregulation of these mechanisms contributes to the pathogenesis of neurodegenerative and neurodevelopmental disorders, including Alzheimer’s disease, Parkinson’s disease, and amyotrophic lateral sclerosis. In the nervous system, lncRNAs: Modulate neuronal differentiation and synaptic plasticity; Participate in neuroinflammation and are implicated in neurodegenerative and neurodevelopmental diseases [1,85]. BACE1-AS, an antisense transcript of the β-secretase gene, stabilizes BACE1 mRNA and enhances amyloid β production in Alzheimer’s disease [86]. Dysregulation of specific lncRNAs contributes to Alzheimer’s disease (AD), Parkinson’s disease (PD), Amyotrophic lateral sclerosis (ALS), Huntington’s disease (HD), Multiple sclerosis (MS), and Autism spectrum disorder (ASD) [87].

Key mechanisms by which lncRNAs contribute to neurological disorders include: Acting as competing ceRNAs that sponge miRNAs, epigenetically silencing or activating neuronal genes, regulating protein aggregation and oxidative stress, and modulating inflammatory pathways [87]. Some notable lncRNAs and their associated neurological diseases are given in Table 2 [60,88,89]. In neurodegenerative diseases, LncRNAs such as *NEAT1*, *MALAT1*, and *HOTAIR* have been shown to modulate amyloid-β aggregation, tau hyperphosphorylation, and α-synuclein accumulation—hallmarks of Alzheimer’s and Parkinson’s disease, respectively [90,91,92]. Similarly, in ischemic stroke, LncRNAs including *SNHG12* regulate apoptotic cascades and blood–brain barrier integrity, positioning them as potential neuroprotective targets [93]. LncRNA MEG3 downregulation was found to be linked with brain lesion and increased angiogenesis after ischemic stroke [94]. In the context of psychiatric disorders such as schizophrenia and major depressive disorder, aberrant LncRNA expression has been linked to disrupted dopaminergic and serotonergic signaling pathways [95,96]. Collectively, these findings highlight LncRNAs as multifunctional regulators at the intersection of epigenetic control and neurological homeostasis, warranting further mechanistic investigation to translate their clinical relevance into viable therapeutic strategies [2].

LncRNAs are key regulators of neuronal health and disease, offering Insights into disease mechanisms, Potential diagnostic biomarkers, and Novel therapeutic targets.

### 5.4. Immune and Inflammatory Diseases

In the immune system, lncRNAs are now recognized as critical regulators of: Immune cell development and differentiation; Cytokine production; Inflammatory signaling pathways (e.g., NF-κB, JAK/STAT); Autoimmune responses. LncRNAs such as NEAT1 and THRIL modulate inflammatory gene expression and have roles in autoimmune diseases like rheumatoid arthritis and systemic lupus erythematosus [105]. In Rheumatoid Arthritis (RA): NEAT1 promotes synovial fibroblast proliferation and pro-inflammatory cytokine production; HOTAIR upregulates MMPs contributing to joint destruction; MEG3 shows anti-inflammatory roles by inhibiting NF-κB activation [106]. In Systemic Lupus Erythematosus (SLE): GAS5 is downregulated in SLE patients and negatively regulates T-cell proliferation; linc0949 is decreased in active SLE and correlates with disease activity [107]. In Inflammatory Bowel Disease (IBD): DQ786243 enhances NF-κB pathway activation in ulcerative colitis; LncRNA IFNG-AS1 promotes Th1 cell differentiation in Crohn’s disease. In Multiple Sclerosis (MS): MALAT1 is overexpressed in MS patients, promoting neuroinflammation; LncRNA PVT1 enhances Th17 differentiation [108]. LncRNAs regulate immune and inflammatory processes by: Sponging microRNAs (ceRNA mechanism) (e.g., NEAT1/miR-129-5p/TLR4 axis); Epigenetic remodelling (e.g., recruiting histone methyltransferases); Transcriptional regulation of cytokines and chemokines; Modulation of signaling pathways (e.g., NF-κB, JAK/STAT). LncRNAs are useful for several therapeutic implications, such as Biomarkers (e.g., GAS5, NEAT1 for diagnosis or monitoring immunological responses), Targeted therapies: Antisense oligonucleotides to modulate pathogenic lncRNAs, and Immune modulation: Restoring homeostatic lncRNA expression to suppress autoimmunity [109]. LncRNAs are integral regulators of immunity and inflammation. Ongoing research continues to uncover their diagnostic and therapeutic promise in immune-mediated diseases.

## 6. Therapeutic Targeting of lncRNAs in Diseases

Despite increasing interest in targeting lncRNAs for therapeutic purposes, several critical challenges limit their clinical translation. Efficient and tissue-specific delivery remains a major barrier, particularly for organs beyond the liver and central nervous system. In addition, many functionally important lncRNAs are localized in the nucleus, restricting the effectiveness of RNA interference-based strategies and necessitating alternative approaches such as antisense oligonucleotides or gapmers. Isoform complexity further complicates therapeutic design, as multiple transcript variants from a single locus may have distinct or even opposing functions. Moreover, the lack of reliable pharmacodynamic biomarkers hampers the ability to assess target engagement and therapeutic efficacy in vivo. Finally, off-target effects and unintended perturbation of regulatory networks remain significant concerns, particularly given the extensive interactions of lncRNAs with chromatin, proteins, and other RNAs [110]. Addressing these challenges is essential for advancing lncRNA-based therapeutics toward clinical application. Beyond these disease contexts, recent evidence indicates that lncRNAs mediated mitochondrial gene regulation is critically involved in the pathogenesis of male [111], which opens a broad view of therapeutic implications of lncRNA. From a translational perspective, enthusiasm for lncRNA targeting must be balanced against substantial unresolved challenges. These include tissue-specific delivery, on-target versus off-target effects, nuclear accessibility, isoform complexity, and the difficulty of identifying pharmacodynamic biomarkers that confirm target engagement. Moreover, therapeutic relevance depends not only on whether a lncRNA is dysregulated, but also on whether its function is causally required for disease maintenance and can be modulated safely in vivo. As a result, only a limited number of lncRNAs currently meet the threshold for serious translational prioritization. Outlined below are some major areas of therapeutic relevance.

### 6.1. LncRNAs as Biomarkers

Due to their tissue specificity and stability in bodily fluids, lncRNAs have emerged as diagnostic and prognostic biomarkers, like HULC and UCA1, which are being explored as diagnostic and prognostic biomarkers in cancers [112]. LncRNAs like PCA3 serve as biomarkers in prostate cancer. LncRNAs are also used as prognostics, like Elevated HOTAIR and MALAT1 correlate with poor survival. LIPCAR and MIAT are linked to heart failure prognosis. BACE1-AS correlates with Alzheimer’s pathology [113]. However, the clinical utility of lncRNA-based biomarkers depends on the availability of robust and reproducible detection methods, as well as standardized thresholds across patient populations. In addition, variability in expression across tissues and disease stages may limit their generalizability.

### 6.2. LncRNA-Targeted Therapies

Several lncRNA-targeted therapies have advanced into preclinical and early-phase clinical trials, including antisense inhibitors (e.g., targeting MALAT1, HOTAIR, NEAT1, etc., for cancers). LncRNA mimics to restore tumor suppressor functions and Nanoparticle delivery systems for targeted delivery. Approaches include ASOs, RNA interference (RNAi), and CRISPR/Cas9 to knock down or edit disease-associated lncRNAs. Gapmers, for example, have shown efficacy in silencing MALAT1 in vivo [114]. Silencing oncogenic lncRNAs sensitizes tumours to chemotherapy. CRISPR-Cas9 and CRISPR-dCas platforms are being developed to selectively repress or activate lncRNAs, offering precise modulation. Notable advances include: CRISPRi-mediated silencing of oncogenic lncRNAs and CRISPRa-driven overexpression of tumor suppressive lncRNAs [46]. Recent breakthroughs in ASOs, small interfering RNAs (siRNAs), gapmeRs, and CRISPR/Cas-based technologies have greatly expedited the development of lncRNA-targeted therapeutics (Table 3). Despite their promise, these approaches face challenges related to efficient delivery, particularly to non-hepatic tissues, as well as potential off-target effects arising from partial sequence complementarity and immune activation. CRISPR-based strategies offer locus-specific targeting; however, they may disrupt regulatory DNA elements or transcriptional activity independent of the RNA transcript, complicating interpretation and raising safety concerns.

#### 6.2.1. Translational Feasibility and Therapeutic Targeting of lncRNAs

Although lncRNAs represent promising therapeutic targets due to their regulatory roles and tissue-specific expression patterns, successful clinical translation depends on overcoming several practical barriers. Unlike protein targets, lncRNAs often function through complex RNA–DNA–protein interactions and exhibit cell-type-specific expression, nuclear localization, and isoform diversity. Consequently, therapeutic development must consider not only the availability of targeting technologies but also key factors such as delivery efficiency, transcript localization, specificity, and reliable biomarkers of target engagement. Organizing therapeutic strategies around these translational constraints provides a more realistic framework for evaluating the feasibility of lncRNA-targeted interventions.

#### 6.2.2. Delivery Challenges and Tissue Specificity

Efficient delivery remains one of the major obstacles for lncRNA-targeted therapeutics. Many lncRNAs exhibit highly tissue-specific or cell-type-specific expression patterns, which necessitates targeted delivery systems capable of reaching relevant tissues while minimizing systemic exposure. ASOs and siRNAs have shown promising delivery profiles in certain tissues such as liver and central nervous system; however, delivery to other organs, including solid tumors and cardiovascular tissues, remains challenging. Nanoparticle-based carriers, lipid nanoparticles, and ligand-conjugated oligonucleotides are currently being explored to enhance tissue targeting and cellular uptake. Another critical barrier is endosomal escape, as a substantial fraction of delivered RNA therapeutics becomes trapped in endosomal compartments before reaching the cytoplasm or nucleus. These limitations highlight the need for advanced delivery platforms capable of achieving tissue specificity, efficient cellular uptake, and endosomal escape.

#### 6.2.3. Nuclear Versus Cytoplasmic lncRNA Targeting

The subcellular localization of lncRNAs strongly influences therapeutic strategy selection. Many functionally characterized lncRNAs, including XIST, NEAT1, and MALAT1, primarily reside in the nucleus where they regulate chromatin organization or transcriptional programs. Nuclear localization limits the effectiveness of RNA interference mechanisms, which predominantly operate in the cytoplasm. In contrast, antisense oligonucleotides and gapmer-based strategies can efficiently degrade nuclear transcripts via RNase H–mediated cleavage, making them more suitable for targeting nuclear lncRNAs. Consequently, selecting the appropriate therapeutic platform requires careful consideration of transcript localization and accessibility within the cell. Thus, subcellular localization must be carefully considered when selecting therapeutic strategies, as mismatched approaches may result in reduced efficacy.

#### 6.2.4. On-Target Versus Off-Target Toxicity

Another critical challenge is distinguishing between intended target inhibition and unintended off-target effects. Because lncRNAs frequently interact with multiple protein complexes or chromatin regions, perturbation of a single transcript may have widespread downstream consequences. Additionally, oligonucleotide-based therapeutics can produce sequence-dependent or chemistry-dependent off-target effects. These include hybridization to unintended transcripts, activation of innate immune responses, or nonspecific binding to proteins. Rigorous experimental validation, including transcriptome-wide analysis and multiple independent perturbation approaches, is therefore essential to confirm target specificity and minimize toxicity risks. Given the extensive interaction networks of lncRNAs, even on-target perturbation may produce unintended downstream effects, underscoring the need for comprehensive safety evaluation.

#### 6.2.5. Isoform Complexity and Transcript Specificity

Many lncRNA loci produce multiple alternatively spliced isoforms with distinct functional properties. For example, NEAT1 generates two major isoforms that play different roles in paraspeckle formation. Therapeutic strategies targeting only a single isoform may therefore produce incomplete or unintended biological effects. Designing interventions that discriminate between isoforms, while avoiding disruption of neighboring regulatory elements within the same genomic locus, represents a significant technical challenge. Advances in long-read sequencing and isoform-specific targeting technologies may help address this issue. Isoform-specific targeting remains a significant challenge, as different transcript variants may have distinct or even opposing biological functions.

#### 6.2.6. Pharmacodynamic Biomarkers and Target Engagement

Demonstrating target engagement remains a major hurdle in the clinical development of RNA-based therapeutics. For protein-targeted drugs, biomarker readouts are often based on measurable changes in protein activity or downstream signaling. In contrast, assessing the effectiveness of lncRNA-targeted therapies requires reliable pharmacodynamic biomarkers that reflect transcript depletion or functional disruption. Such biomarkers may include changes in downstream gene expression programs, chromatin states, or disease-associated molecular signatures. Developing robust biomarkers is therefore critical for evaluating therapeutic efficacy in both preclinical and clinical studies. The absence of reliable pharmacodynamic biomarkers further limits the ability to monitor therapeutic response and confirm target engagement in clinical settings.

#### 6.2.7. Current Therapeutic Platforms for lncRNA Targeting

Several molecular technologies have been developed to modulate lncRNA activity, including antisense oligonucleotides, RNA interference strategies, and CRISPR-based transcriptional modulation systems. Among these approaches, antisense oligonucleotides and gapmer technologies have shown particular promise for targeting nuclear lncRNAs due to their ability to recruit RNase H and induce transcript degradation. RNA interference approaches are more effective for cytoplasmic lncRNAs, whereas CRISPR interference (CRISPRi) and CRISPR activation (CRISPRa) systems enable locus-specific repression or activation at the transcriptional level. While these platforms provide powerful tools for manipulating lncRNA expression, their clinical feasibility ultimately depends on overcoming the delivery, specificity, and biomarker challenges described above. Ultimately, the clinical success of these platforms will depend on their ability to overcome delivery, specificity, and biomarker-related limitations.

### 6.3. Epigenetic and Chromatin-Regulating lncRNAs

#### 6.3.1. HOTAIR

HOTAIR is a well-studied oncogenic lncRNA that induces cancer development and metastasis via epigenetic remodelling. LncRNA HOTAIR is located in a region between HOX11 and HOX12 on chromosome 12q13.3, with 2.2 kb nucleotides length size. HOTAIR interacts with Polycomb Repressive Complex 2 (PRC2) and Lysine-Specific Demethylase 1 (LSD1) complexes via 5′-3′ domains, leading to the HOXD gene cluster and other genes, causing histone H3K27 trimethylation and transcriptional suppression of tumor suppressor genes. Several studies reported that HOTAIR is generally expressed in breast cancer, colon cancer, and liver cancer tissues and plays a crucial cancer cell proliferation and inhibition of oncogenes [136,137]. Preclinical investigations with ASOs and siRNA-mediated silencing of HOTAIR showed considerable reduction in tumor growth and invasion, underlining its therapeutic potential [47]. A study involving 148 patients observed that HOTAIR have significant diagnostic value for detecting the pathological staging of non-small cell lung cancer with high specificity (86.9%) and sensitivity (52.3%) (Yao et al., 2022) [138]. In another study, HOTAIR was reported to show 0.9723 area under the curve (AUC) with 92% sensitivity and 98% specificity for vaginal discharge of cervical cancer patients, which indicates HOTAIR is a potential diagnostic tool for cervical cancer (Liu et al., 2023) [139]. HOTAIR may be a potential therapeutic target for CNS disorders because in vitro and in vivo studies have demonstrated that inhibition of HOTAIR expression can play a therapeutic role in CNS disorders through multiple mechanisms [87]. Overall, these studies indicate that HOTAIR has strong potential as a diagnostic biomarker in cancer and may also be explored in the context of other disease-related research.

The functional role of HOTAIR is supported by multiple lines of evidence, including in vitro perturbation studies (siRNA/ASO-mediated knockdown), chromatin interaction assays (e.g., RNA immunoprecipitation and chromatin isolation techniques), and in vivo validation in mouse xenograft models, placing it within a moderate-to-high evidence tier. However, some studies suggest that observed phenotypes may be influenced not only by the HOTAIR transcript itself but also by regulatory elements within the HOXC locus [140]. Therefore, while HOTAIR is widely considered a key epigenetic regulator, distinguishing RNA-dependent effects from locus-dependent regulatory activity remains an important area of ongoing investigation.

#### 6.3.2. ANRIL

ANRIL, alternatively referred to as CDKN2B-AS1, is a 3.8 kb long noncoding RNA transcribed in the antisense orientation of this gene cluster [141]. ANRIL regulates gene expression at the post-transcriptional level by acting as a competing ceRNA that interacts with miRNAs and proteins. In addition, ANRIL modulates gene expression at the chromatin level, both positively and negatively, by guiding the recruitment of chromatin modifiers or transcriptional activators to specific genomic loci [142]. ANRIL promotes *cis*-acting transcriptional silencing of the CDKN2A and CDKN2B genes by facilitating the recruitment of Polycomb group (PcG) complexes to the 9p21 locus, thereby leading to increased cellular proliferation [142]. A crucial consideration is the potential generation of at least 24 alternatively spliced ANRIL isoforms, several of which show overexpression that correlates with severe and highly prevalent diseases, including coronary artery disease, diabetes, and various cancers [143]. Along with these diseases, a study reported that the ANRIL regulates the CTNNB1 gene-mediated transcriptional regulation against inflammatory response [144].

According to the studies, ANRIL-mediated a plausible therapeutic strategy may involve reducing its intracellular levels within malignant and affected tissues. This could be accomplished using approaches such as RNA interference (RNAi), ASOs, RNA-targeting CRISPR/Cas systems, or epigenetic modulators designed to modify the epigenetic landscape of the ANRIL locus [145]. Utilising small-designed molecules to inhibit the ANRIL functions could also be the best option for ANRIL-mediated therapies [146]. Therapeutic targeting of ANRIL may restore cell cycle regulation and slow disease development, especially in atherosclerosis and cancer [147,148].

#### 6.3.3. XIST

lncRNA XIST is localised at the chromosome inactivation center (XIC) of chromosome Xq13.2 with a set of 15,000–20,000 nt sequences. The XIST plays a key role in X-chromosome inactivation during early embryonic development. Beyond its physiological function, aberrant XIST expression has been increasingly implicated in the pathogenesis of multiple human diseases, including cancers, neurological disorders, cardiovascular diseases, and inflammatory conditions. Dysregulated XIST expression contributes to disease progression through epigenetic remodelling, transcriptional repression, post-transcriptional regulation, and interactions with RNA-binding proteins and microRNAs. According to its multifaceted regulatory capacity, XIST has emerged as both a disease biomarker and a therapeutic target, particularly in conditions where its aberrant overexpression or silencing drives pathological phenotypes.

Several studies indicate that XIST induces bladder cancer development by regulating miRNAs (miR-124, miR-139-5p, miR-200c, miR-133a, and miR-335) or other target genes (Wnt1, TET1, and p53) to control cell growth, migration, invasion and metastasis [149]. These findings indicate that lncRNA XIST may be exploited as a promising therapeutic target and prognostic indicator. Similarly, XIST is reported to facilitate different tumor development, such as pituitary neuroendocrine tumor, neuroblastoma, thyroid cancer, colon cancer, renal cell carcinoma, and prostate cancer [150,151,152]. Targeted inhibition of XIST can be an effective therapeutic option to inhibit tumor development in these types of cancer. A study reported that the knockdown of XIST significantly inhibits colorectal cancer cell proliferation, invasion, epithelial–mesenchymal transition and stem cell formation in vitro as well as tumor growth and metastasis in vivo. Further, this study also showed that the Knockdown of XIST significantly reduced the expression of the ZEB 1 gene, which directly targets miR-200b-3p and suppresses tumor growth [153]. Also, XIST is abnormally expressed in many sex-biased diseases. Genetic manipulation of XIST was reported to inhibit the prognosis of many sex-biased diseases in animal models [84], which suggests that XIST can be a potential therapeutic target in different diseases.

### 6.4. Nuclear Structure and Transcriptional Control lncRNAs

#### 6.4.1. MALAT1

MALAT1 controls alternative splicing, transcription, and EMT. MALAT1 overexpression is linked to metastasis and poor survival in lung, breast, and colorectal malignancies. GapmeR-mediated MALAT1 knockdown significantly reduces metastasis in vivo without causing significant damage, highlighting its translational potential [154]. Transcriptional factor specificity protein 1 (SP1) has been reported to overexpressed in many types of cancers. In a study of lung adenocarcinoma cells, MALAT 1 was found to interact with SP1 protein and promote SP1-mediated transcriptional regulation of SP1 target genes [155], which indicates targeting by knockdown of MALAT, may open a path for the therapeutic strategy against lung adenocarcinoma. Similarly, lncRNA-MALAT1 was reported in non-small cell lung cancer cells (NSCLC) progression by targeting miR-202 [156,157]. Also, targeting the MALAT1/miR-202/Gli2-mediated regulatory pathway could be a novel therapeutic strategy for gastric cancer [158]. Silencing MALAT1 suppresses gastric cancer cell proliferation and induces apoptosis by upregulating miR-22-3p and downregulating ErbB3 expression. Silencing study of the MALAT1 in the inhibition of tumour growth in mice may also be a promising indication of a therapeutic target for gastric cancer [159]. Not only for cancer but also for HIV-1, MALAT1 showed a significant role in the infection. MALAT1 ASOs are reported to suppress HIV-1 p24 production and HIV-1 Gag gene expression and decreased expression of miR-155 and SOCS1, as well as the production of proinflammatory cytokines by HIV-1-infected macrophages. These studies suggest a potential role for targeting MALAT1 as a therapeutic approach to eliminate HIV-1 reservoirs [160], and other regulating pathways of the disease.

MALAT1 represents one of the most extensively studied lncRNAs, with functional evidence derived from in vitro knockdown experiments, ASO approaches, and multiple in vivo models, including genetic knockout mice [154,161]. These studies collectively place MALAT1 in a high evidence tier with well-supported roles in transcriptional regulation, alternative splicing, and metastasis. Notably, MALAT1 knockout models have demonstrated context-dependent phenotypic effects, suggesting that its functional relevance may vary across biological systems. Importantly, most evidence supports a predominantly RNA-dependent mechanism, although subtle contributions from transcriptional activity at the locus cannot be completely excluded.

#### 6.4.2. NEAT1

NEAT1 is a structural component of nuclear paraspeckles that is essential for stress responses, immunological signaling, and oncogenesis. Prostate cancer, glioma, and neurodegenerative diseases have all shown elevated NEAT1 expression. NEAT1 knockdown via ASO affects paraspeckle formation and lowers tumor growth, making it an attractive therapeutic target [162]. NEAT1 can inhibit apoptosis and encourage chemoresistance in cancer cells by regulating the mitochondrial apoptosis pathway. In a study, NEAT1 was reported to inhibit apoptosis and induce paclitaxel resistance by reducing the expression of apoptosis-related proteins, cleaved caspase3 and cleaved PARP, which consequently activated the AKT/mTOR signaling pathway [163,164]. However, in ovarian cancer cells, NEAT1 was reported to upregulate the expression of PARP1 and simultaneously inhibit BAX expression and c-caspase3 by targeting miR-770-5p, which inhibits apoptosis and induces cisplatin [165]. Also, NEAT1 directly interacts with AKAP8/PKA to activate Hippo signaling for self-renewal of liver cancer stem cells [166]. Additionally, in gastric cancer patients, NEAT1 was reported to be associated with radiotherapy resistance and adverse prognosis [167]. Not only in cancer, but also in corneal neovascularization, NEAT1 was reported to promote inflammatory response [168]. In a recent study, Adeno-associated virus-mediated *Neat1* knockdown in combination with F8 gene augmentation showed potential effect against haemophilic joint disease [169]. Overall, studies indicate that targeting NEAT1 knockdown and other options, such as RNA sponging, chemical inhibitors, etc., could be a promising option for targeting related diseases.

The role of NEAT1 is supported by strong experimental evidence, including in vitro perturbation studies, structural and imaging analyses of paraspeckle formation, and in vivo genetic models, placing it in a high evidence tier. NEAT1 has been shown to function as an architectural RNA essential for paraspeckle assembly, with clear links between its expression and cellular stress responses and disease phenotypes [170]. In this case, the functional effects are largely attributed to the RNA transcript itself, particularly its structural role in nuclear organization, rather than to the genomic locus. However, isoform-specific functions (e.g., NEAT1_1 vs. NEAT1_2) add an additional layer of complexity that requires careful interpretation.

#### 6.4.3. PVT1

PVT1 is typically co-amplified with the MYC oncogene, and it contributes to carcinogenesis by stabilizing MYC protein and activating carcinogenic transcriptional pathways. PVT1 encodes an oncogenic lncRNA; however, recurrent translocations and deletions in human cancers indicate alternative mechanisms. A study demonstrates that the PVT1 promoter exerts a tumor-suppressive function independent of the PVT1 lncRNA transcript. CRISPR interference targeting the PVT1 promoter enhances breast cancer cell competitiveness and tumor growth in vivo [171]. This promoter competition regulates MYC transcriptional pause release in a cis-acting, allele-specific manner, as PVT1 exhibits developmentally regulated monoallelic expression. Cancer genome sequencing has identified recurrent mutations within the PVT1 promoter, and functional genome-editing studies confirm that disruption of this promoter promotes oncogenic growth. Collectively, these findings underscore lncRNA gene regulatory elements, particularly promoters, as critical disease-associated DNA elements [171]. A recent study investigated the combined effects of siRNA-mediated PVT1 silencing and paclitaxel treatment on gastric cancer (AGS) cells. PVT1 knockdown significantly reduced cell viability, inhibited migration, and enhanced paclitaxel-induced apoptosis, accompanied by increased G2/M cell-cycle arrest. These findings suggest that targeting lncRNA PVT1 may potentiate chemotherapeutic efficacy in gastric cancer [65]. Using an ex vivo metanephric organ culture (MOC) model of autosomal dominant polycystic kidney disease (ADPKD), Eckberg et al. (2022) [172] demonstrated that shRNA-mediated knockdown of the lncRNA PVT1, which is upregulated in PKD1/PKD2-mutant kidneys and in patients with ADPKD, significantly reduced cyst formation in both wild-type and Pkd1-null MOCs. Mechanistically, PVT1 suppression decreased c-MYC protein levels without altering MYC mRNA, indicating post-transcriptional regulation. These findings identify PVT1 as a critical modulator of cystogenesis and a potential therapeutic target in ADPKD. Emerging evidence highlights lncRNA PVT1 as a key therapeutic target across multiple cancer types. In estrogen receptor α (ERα)-positive breast cancer, PVT1 facilitates anti-estrogen resistance by mediating the functional interaction between EZH2 and ERα; thus, disruption of this complex through PVT1 inhibition may effectively suppress resistant tumor cell proliferation. Given the established involvement of both PVT1 and EZH2 in therapy resistance, ASO-based silencing of PVT1 represents a promising pharmacological strategy, either alone or in combination with standard treatments [173,174,175,176]. Similarly, in hepatocellular carcinoma, PVT1 is transcriptionally activated by the TGF-β1/Smad3 signaling pathway and promotes tumor proliferation and metastasis via distinct ceRNA-mediated axes, namely, miR-186-5p/Smad6 and miR-143-3p/NRG1. These findings further support PVT1 as a context-dependent oncogenic regulator and a potential therapeutic target in HCC [177].

## 7. Emerging Technologies in lncRNA Research

Recent advances in high-resolution genomic and transcriptomic technologies have significantly transformed the study of lncRNAs, enabling more precise characterization of their expression, localization, and functional roles. Single-cell RNA sequencing (scRNA-seq) has revealed substantial cell-type-specific heterogeneity in lncRNA expression, uncovering previously unrecognized regulatory roles in development and disease that are masked in bulk transcriptomic analyses [178]. Complementary to this, spatial transcriptomics approaches now allow the mapping of lncRNA expression within intact tissue architecture, providing critical insights into their spatial organization and context-dependent functions in complex biological systems.

In parallel, advances in RNA structure–function mapping techniques, including SHAPE-MaP, icSHAPE, and crosslinking-based approaches, have begun to elucidate the structural features that underlie lncRNA interactions with proteins, DNA, and other RNAs [179]. These studies are revealing that secondary and tertiary RNA structures are key determinants of lncRNA function, influencing binding specificity and regulatory activity.

From a translational perspective, recent progress in RNA-targeting therapeutics has accelerated the clinical development of lncRNA-based interventions. ASOs, gapmers, and RNA interference strategies targeting disease-associated lncRNAs are now being evaluated in preclinical and early clinical studies, particularly in cancer and neurological disorders. These developments highlight the growing feasibility of modulating lncRNA activity in vivo and underscore the importance of integrating mechanistic insights with therapeutic innovation.

Together, these emerging technologies are reshaping the lncRNA research landscape by enabling more accurate functional annotation, improving reproducibility, and facilitating the translation of basic discoveries into clinical applications.

## 8. Current Challenges and Future Perspectives in LncRNA-Mediated Disease Therapies

Challenges include understanding lncRNA structure-function relationships, off-target effects, and efficient delivery systems. However, the expanding catalogue of functional lncRNAs and therapeutic platforms holds great promise. As a future perspective, wide expansion of lncRNAs can be done, such as the development of lncRNA-targeted ASOs, Integration of lncRNA expression profiling into precision oncology, and Elucidation of lncRNA–protein complexes to uncover new vulnerabilities. Despite the promise, several challenges remain, such as Specificity (Off-target effects can compromise safety); Delivery (Efficient in vivo delivery is still a barrier); Functional Annotation (Many lncRNAs remain uncharacterized); and Regulatory Considerations (Therapeutic approvals require robust validation) [180].

The disease-specific expression patterns and functional impact of ceRNA networks make miRNA-sponging lncRNAs promising therapeutic candidates. ASOs, small molecule inhibitors, and CRISPR-based approaches have the potential to modulate lncRNA levels or disrupt pathogenic lncRNA-miRNA interactions [181]. However, therapeutic translation faces several challenges, including off-target effects, delivery to specific tissues, and compensatory network dynamics that may mitigate single-node targeting. Despite encouraging preclinical results, lncRNA-targeted therapeutics have numerous hurdles, including effective tissue-specific transport, off-target effects, and insufficient functional annotation. The continued integration of multi-omics methods and sophisticated delivery technologies is likely to speed up the clinical translation of lncRNA-based therapies.

Despite substantial progress in lncRNA biology, the field continues to face important conceptual and methodological challenges. First, the strength of functional evidence is highly uneven. While a subset of lncRNAs has been investigated through detailed mechanistic and genetic studies, many proposed functions remain based on differential expression, correlation analyses, or limited knockdown experiments. This creates a gap between the large number of reported disease-associated lncRNAs and the smaller number of lncRNAs with reproducible, causally supported functions. Second, reproducibility remains a major concern. Different experimental approaches, including RNA interference, antisense oligonucleotides, CRISPR interference, and genomic deletion, do not always produce concordant results. In some cases, phenotypes attributed to the RNA transcript may instead reflect effects of the underlying DNA locus, promoter activity, transcriptional interference, or neighboring regulatory elements. Distinguishing transcript-dependent from locus-dependent functions is therefore essential for accurate mechanistic interpretation. Third, lncRNA activity is often highly context dependent. Many lncRNAs exhibit cell-type-specific, developmental-stage-specific, or stress-responsive expression, and their interacting partners may vary across biological settings. As a consequence, a mechanism established in one model system may not be generalizable to another. This context dependence is particularly relevant in disease studies, where observations from transformed cell lines or single cohorts may not reflect broader biological or clinical reality.

A major challenge in lncRNA research is the variability in functional outcomes observed across different experimental approaches. RNAi and ASOs are widely used to reduce lncRNA transcript levels; however, both methods are susceptible to off-target effects, including unintended hybridization with partially complementary RNAs and activation of innate immune responses. In contrast, CRISPR-based approaches enable locus-level perturbation through genomic deletion, CRISPRi, or activation CRISPRa, but these strategies may disrupt regulatory DNA elements, transcriptional activity, or chromatin architecture independent of the RNA transcript itself. Consequently, discrepancies between RNAi/ASO-mediated knockdown and CRISPR-based perturbations are frequently observed, complicating the interpretation of lncRNA function.

In addition, many lncRNA loci produce multiple alternatively spliced isoforms with distinct expression patterns and functional properties. Perturbation strategies that do not discriminate between isoforms may therefore lead to incomplete or misleading conclusions. Furthermore, lncRNA functions are often highly cell-type- and context-specific, and results obtained in a particular model system may not be generalizable across biological conditions. These factors highlight the importance of integrating multiple complementary approaches, including transcript-specific targeting, locus-resolved genetic analyses, and in vivo validation, to establish robust and reproducible mechanistic insights. Careful experimental design and critical interpretation are therefore essential to distinguish true functional roles from methodological artifacts.

Finally, several important questions remain unresolved. For many lncRNAs, it is still unclear which structural features determine function, how isoform diversity affects biological activity, and which molecular interactions are required under physiological conditions. At the translational level, improved strategies are needed to define which lncRNAs are truly actionable as biomarkers or therapeutic targets. Addressing these issues will require more rigorous functional validation, quantitative mechanistic studies, and integration of single-cell, spatial, and locus-resolved approaches.

## 9. Conclusions

LncRNAs have emerged as pivotal regulators of gene expression and cellular homeostasis, exerting their functions through diverse molecular mechanisms, including chromatin remodeling, transcriptional modulation, and post-transcriptional regulation. Their dysregulation is now recognized as a key contributor to the pathogenesis of a wide range of diseases, including cancer, neurodegenerative disorders, and cardiovascular conditions. Increasing evidence also highlights the potential of lncRNAs as diagnostic biomarkers, prognostic indicators, and therapeutic targets, supported by advances in high-throughput sequencing, molecular profiling, and functional genomics that have revealed their complexity and strong tissue specificity.

However, despite these advances, significant challenges remain in translating lncRNA discoveries into clinical applications, particularly with respect to specificity, delivery, and safety of lncRNA-targeted interventions. lncRNAs represent a rapidly expanding class of regulatory molecules with significant implications for human health and disease. Despite substantial progress in characterizing their molecular functions, a large proportion of lncRNAs remain poorly understood, highlighting a critical need for systematic and scalable approaches to functional annotation. Future research should prioritize distinguishing RNA-dependent effects from locus-dependent regulatory mechanisms using precise, locus-resolved perturbation strategies, including CRISPR-based and transcript-specific targeting approaches.

Advances in high-resolution technologies, such as single-cell and spatial transcriptomics, are expected to further refine our understanding of the cell-type-specific and context-dependent roles of lncRNAs. In parallel, emerging RNA structure–function mapping techniques will be essential for elucidating how structural features govern lncRNA interactions and regulatory activity. Integrating these approaches with quantitative and reproducible experimental frameworks will be crucial for establishing robust mechanistic models.

From a translational perspective, the development of reliable pharmacodynamic biomarkers and improved delivery systems remains a major priority for enabling clinical applications of lncRNA-targeted therapies. Identifying which lncRNAs are truly actionable, based on causal roles in disease and therapeutic tractability, would be essential for advancing precision medicine strategies. Ultimately, bridging the gap between mechanistic insight and clinical implementation will require coordinated efforts combining functional genomics, advanced technologies, and clinically relevant model systems.

## Figures and Tables

**Table 2:** Some notable lncRNAs and their associated neurological disorders.

LncRNAS	Associated Disorders	Functions	References
BACE1-AS	Alzheimer’s disease	Stabilizes BACE1 mRNA → ↑ Aβ production	[97]
NEAT1	ALS, AD, PD	Paraspeckle formation, neuroinflammation	[98]
MALAT1	Stroke, AD, PD	Synaptic plasticity, apoptosis regulation	[99]
SNHG1	Parkinson’s disease	miR-15b-5p sponge, modulates α-synuclein	[100]
MEG3	Alzheimer’s disease	p53 activation, pro-apoptotic roles	[101]
LINC-PINT	Alzheimer’s disease	Neuroprotective transcriptional regulation	[102]
GAS5	Ischemic stroke	Promotes neuronal apoptosis	[103]
SOX2-OT	Autism spectrum disorder	Regulates neuronal differentiation	[104]

**Table 3:** lncRNA-mediated therapeutic strategies in different disease conditions.

IncRNA	Diseases	Mechanism/Functional Role	Clinical Relevance/Biomarker	Therapeutic Strategies	References
HOTAIR	Breast cancer, others	Epigenetic regulation via PRC2 recruitment; alters chromatin state → promotes metastasis	Overexpressed in metastatic cancers; predictive of poor prognosis	siRNA/antisense oligo inhibition	[115,58]
MALAT1	Lung cancer, colorectal, diabetes complications	Regulates splicing, transcription; modulates EMT, cell migration	High expression correlates with metastasis and poor survival	GapmeRs/antisense therapies	[116,117]
H19	Hepatocellular carcinoma, bladder cancer	Acts as miRNA sponge, modulating IGF2/H19 imprinting; pro-tumorigenic effects	Elevated in tumor tissue and circulation	RNA inhibitors; CRISPR targeting	[118]
XIST	Various cancers; sex-linked disorders	X-chromosome inactivation; interacts with chromatin modifiers	Dysregulated XIST linked to tumor progression	Targeted silencing modulation	[119]
NEAT1	Prostate cancer, neurodegeneration	Scaffold for paraspeckle formation; influences apoptosis and immune response	Elevated in prostate cancer and ALS	ASOs/antisense inhibition	[120,121]
PVT1	Cancer (leukemia, breast)	Competes with MYC enhancers; regulates oncogenic signaling	Correlates with MYC amplification and poor outcome	Targeted silencing	[122]
GAS5	Breast cancer, inflammation	Decoy for glucocorticoid receptor; induces apoptosis	Low levels associated with cancer progression	Gene therapy to restore expression	[123]
ANRIL	Atherosclerosis, diabetes, cancers	Recruits PRC1/PRC2 to regulate CDKN2A/B; affects cell proliferation	SNPs in ANRIL linked to CVD risk	Small molecule/RNA interference	[124]
TUG1	Retinopathy, cancer	Modulates cell proliferation and apoptosis	Elevated in diabetic retinopathy	Antagomir/RNA targeting	[125]
UCA1	Bladder cancer, ovarian cancer	miRNA sponge; affects drug resistance (e.g., cisplatin)	Diagnostic marker in urine for bladder cancer	RNA inhibitors	[126]
CCAT1	Colorectal cancer	Enhances MYC expression via chromatin looping	Early detection marker in CRC	Antisense/CRISPR inhibition	[127]
FENDRR	Cardiac malformations	Modulates epigenetic regulators (PRC2) & transcription	Linked to congenital heart disease	Gene modulation	[128]
LINC-PINT	Glioma, cancer progression	p53-regulated; interacts with PRC2 to suppress proliferation	Low expression → poor prognosis	RNA replacement therapy	[129]
TERRA	Telomere biology, aging	Regulates telomeric chromatin & telomerase activity	Potential aging/cancer marker	TERRA modulation	[130]
HULC	Hepatocellular carcinoma	Acts as miRNA sponge; promotes tumor growth	High expression in HCC; early diagnosis	ASO inhibition	[131]
lnc-RNA-p21	p53 pathway, cancer	Represses transcription of p53 targets; pro-apoptotic	Correlates with radio-/chemo-response	Gene delivery/modulation	[26]
MEG3	Pituitary tumors, neurological disorders	Tumor suppressor; modulates p53 function	Low in tumors; prognostic marker	Gene therapy	[132]
BDNF-AS	Neurodegenerative diseases	Antisense regulator of BDNF; affects neuronal survival	Biomarker for neuro disorders	ASOs	[133]
LncRNA-LET	Hypoxia-related cancers	Suppressor of NF90; affects hypoxia response	Low levels → tumor progression	Restore expression	[134]
TINCR	Epidermal differentiation, cancer	Post-transcriptional stabilization of mRNAs	Marker in skin disorders	RNA modulation	[135]

## Data Availability

No new data were created; only published data were used for the review article.

## Abbreviations

The following abbreviations are used in this manuscript:

lncRNAs	Long non-coding RNAs
ceRNAs	Endogenous RNAs
lincRNAs	Long-Intergenic lncRNAs
eRNAs	Enhancer-associated long non-coding RNAs
TERC	Telomerase RNA
PRC2	Polycomb repressive complex 2
DNMT1	DNA methyltransferases
MREs	miRNA response elements
cirRNAs	circular RNAs
HOTAIR	HOX Transcript Antisense RNA
ANRIL	Antisense Noncoding RNA in the INK4 Locus
MIAT	Myocardial Infarction Associated Transcript
ASOs	Antisense oligonucleotides
XIST	X-inactive specific transcript
MALAT1	Metastasis-Associated Lung Adenocarcinoma Transcript 1
NEAT1	Nuclear paraspeckle assembly transcript 1
Onco-IncRNA	Oncogenic lncRNAs
PVT1	Plasmacytoma variant translocation 1
EMT	Epithelial–mesenchymal transition
GAS5	Growth arrest-specific transcript 5
PTENP1	Phosphatase and Tensin Homolog Pseudogene 1
TUG1	Taurine upregulated gene 1
LINC-PINT	lncRNA long intergenic nonprotein-coding RNA, p53-induced transcript
NSCLC	Non-small cell lung cancer cells
